# The Survey of Knee Osteoarthritis in the Population over Age 50 Visited in the Health Bus in Kermanshah, Iran

**DOI:** 10.1155/2021/9809565

**Published:** 2021-11-12

**Authors:** MohammadBagher Shamsi, Ameneh Safari, Ali Soroush, Yahya Safari

**Affiliations:** ^1^School of Allied Medical Sciences, Kermanshah University of Medical Sciences, Kermanshah, Iran; ^2^Esfarayen Faculty of Medical Sciences, Esfarayen, Iran; ^3^Department of Physical Therapy, Kermanshah University of Medical Sciences, Kermanshah, Iran

## Abstract

Along with an aging population worldwide, knee osteoarthritis (KOA), which is the main cause of musculoskeletal pain and disability in the elderly and decreases the quality of life, is prevalent, and their impact is widespread. This study aimed to evaluate the knee osteoarthritis status among the population over age 50 in Kermanshah, Iran. The research community consisted of the population who has been visited in the health bus in Kermanshah in 2016-2017, of which 589 were chosen by an available sampling method. A WOMAC questionnaire was used to determine the prevalence of knee osteoarthritis. The prevalence of knee stiffness rate after sitting, lying down, or resting during the day among women and men were 40.7% and 20.5%, respectively. According to the findings, the highest prevalence rate of knee pain was in subjects with a BMI higher than 30 (31.6%) and BMI 25–30 (24.5%). 39.2% of the subjects never experienced knee pain, 16.6% monthly, 13.4% once a week, 20.4% daily, and 10.4% of them had prolonged knee pain experience. The prevalence of gender-based knee pain was 60.5% among women and 38.6% among men. 30.5% of women and 61.4% of men never experienced knee pain.

## 1. Introduction

Along with an aging population worldwide, the pattern of disease prevalence has changed from acute infectious diseases to chronic noncommunicable diseases such as chronic musculoskeletal disorders. Musculoskeletal disorders are prevalent, and their impact is widespread [1]. They are the most prevalent cause of severe and prolonged pain and physical disabilities, and they have affected hundreds of millions of people worldwide and are considered a global health concern [2, 3].

One of the common musculoskeletal disorders is knee osteoarthritis (KOA), which is the main cause of musculoskeletal pain and disability in the elderly. It decreases the quality of life [4]. This problem often results in severe side effects, and in the second half of the life, it costs the heavy burden of treatment. Knee osteoarthritis includes the degeneration of cartilage with pain inside and around the knee joint, as well as joint stiffness, and decreased range of motion, which ultimately leads to muscle weakness and is the biggest cause of functional disability [4].

Generally, the first symptom of KOA is joint pain, and in patients aged over 55 years, knee pain is often associated with osteoarthritis. Nearly 25% of adults aged over 55 have experienced knee pain at least once a year, which probably is a sign of underlying KOA [4–7].

KOA affects 80% of the elderly and 27 million people in the United States each year, and their treatment costs are $ 185.5 billion annually [6]. The prevalence of this disease is expected to rise the given ever-aging population and the fact that obesity is becoming increasingly common, for example, the prevalence of KOA in Sweden is projected to increase from 13.8% in 2012 to 15.7% in 2032 [5, 8, 9].

The prevalence of KOA in the Asia-Pacific region is 7.50%. This is 5.78% in China, 12.4% in South Korea, 22.0% in rural India, 25.00% in rural population of North Pakistan, and 10.2% in Bangladesh [8, 10–14]. Besides aging and obesity, gender, physical activity level, genetic predisposition, and injury are also risk factors of KOA [4]. Considering that the age-related burden of disease such as osteoarthritis will be significantly accelerated among developing countries, osteoarthritis prevalence will rise, particularly in Asian countries in the future [15].

Mobility and having a painless limb are crucial to perform daily regular activities. The health of the musculoskeletal system is an important part of health. With aging, many issues occur in this motor system. Therefore, particular attention to the physical health and the motor system in a macrolevel society is important as an infrastructure for development. Hence, this study aimed to evaluate the prevalence of KOA as one of the most common problems in the musculoskeletal system among the population over age 50 as an indicator of physical health status in Kermanshah (a city in the west of Iran).

## 2. Materials and Methods

This cross-sectional descriptive study was conducted in 2016-2017. The research community consisted of the population over the age of 50, voluntarily visiting the health bus (a bus that was traveling throughout the city to collect health information) in Kermanshah.

The sample size of the present study calculated 588 subjects based on the WHO-ILAR COPCORD study in Sanandaj [16], with a 95% confidence interval, the accuracy of 4%, and prevalence of 42.8% complaints of musculoskeletal pain in the past 7 days. The inclusion criteria for entering this study were aged over 50 years and the individual's desire to be involved in the study.

The sampling of the present study was carried out in a gradual method until the samples were accomplished. According to the division of urban areas, the residential areas in the city were divided into eight municipal districts, and according to the schedule, each week, the evaluation bus traveled to one of the neighborhoods and was located in each neighborhood for one week.

The bus designed for this purpose had the facilities for collecting data and was located at the centre of the neighborhood, with posters, fliers, and placards announcing that the bus welcomed volunteered participants to be evaluated.

Regardless of having knee pain, those who volunteered to be assessed on the bus, in addition to overall health assessment, their condition of the musculoskeletal system was also assessed, and Western Ontario and McMaster Universities Osteoarthritis Index questionnaire (WOMAC) was completed for them.

Western Ontario and McMaster Universities Osteoarthritis Index questionnaire (WOMAC) was used to assess the status of people in terms of the KOA. The questionnaire includes 17 questions about functional activities, 5 questions about painful activities, and 2 questions about joint stiffness. In the study conducted by Ebrahimzadeh and colleagues in 2014, this questionnaire was translated into Persian, and psychometric evaluation has been performed. In this study, Cronbach's alpha was 0.917, which showed high internal consistency of the questionnaire as a reliable tool. Intercorrelation matrix between different scales of the WOMAC Persian questionnaire version showed a high correlation between the subscales of stiffness, pain, and physical function. In addition, this study showed that the WOMAC Persian index is a valid and reliable clinical tool for reporting KOA [17]. Finally, after completing the questionnaire by a questioner (researcher), the data were analyzed by the SPSS software version 18 and expressed using descriptive statistics.

## 3. Results and Discussion

### 3.1. Results

The results of the recent study showed that about 72% of the participants were women. 67% of the samples were housewives. Furthermore, 35% of them had a BMI over 30 (Table 1).

According to the results of the WOMAC, the prevalence of knee pain among the studied samples was 60.8%. Meanwhile, 39.2% of the subjects never experienced knee pain, 16.6% had monthly, 13.4% once a week, 20.4% on daily basis, and 10.4% of them had prolonged knee pain experience. The results of Section 1 of the knee questionnaire are given in Table 2.

The prevalence of knee pain when doing activities such as going up and downstairs, sitting, and lying down is given in Table 3.

The prevalence of knee stiffness immediately after morning wake-up was 33.3% among participants. Furthermore, the prevalence of knee stiffness after sitting, lying down, or resting during the day was 35% (Table 4).

According to the data, the prevalence of gender-based knee pain was 60.5% among women and 38.6% among men. 30.5% of women and 61.4% of men never experienced knee pain. 19.1% of women and 10.2% of men once a month, 13.0% of women and 14.5% of men once a week, 23.6% of women and 12.0% of men daily, and 13.7% of women and 1.8% of men had prolonged knee pain experience (Table 5).

Table 6 is hyperlinked and could be accessed to address the prevalence of gender-based knee pain through the various activities between men and women.

The findings indicated that the prevalence of knee stiffness immediately after morning wake-up based on gender was 38.3% among women participants and 19.3% among men.

The knee stiffness prevalence rate after sitting, lying down, or resting during the day was 40.7% among women and 20.5% among men.

**Figure 1:**
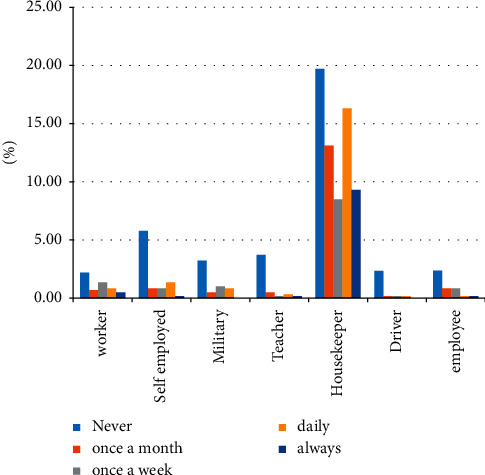
The prevalence of knee pain associated with type of occupation.

According to the findings of the present study, the high prevalence of daily and prolonged knee pain was recognized among housewives (Figure 1).

The results presented in Table 7, hyperlinked and could be read, indicated that according to the findings, the highest prevalence of knee pain was in subjects with BMI higher than 30 (31.6%) and BMI 25–30 (24.5%). The lowest prevalence of knee pain was seen in subjects with lower BMI than 18.5 (1%) (Table 7). The prevalence of knee stiffness associated with the BMI of the studied participants is given in Table 7.

### 3.2. Discussion

Considering the importance of prevention and early treatment of KOA and providing appropriate guidelines in order to prevent it, this study aimed to evaluate the KOA status among the population over age 50 in Kermanshah. The results of the study showed that the prevalence of knee pain in the samples was 60.8%.

The data of the present study indicated that the KOA symptoms increase with age. In a demographic study in urban and rural areas of Bangladesh, the similar findings with age were seen [13]. The results presented in Table 7 provide that the highest prevalence rate of knee pain was in subjects with BMI higher than 30 (31.6%) and BMI 25–30 (24.5%). The lowest prevalence of knee pain was seen in subjects with lower BMIs than 18.5 (1%). The results of Qing Yu and colleagues indicated a strong association between high BMI and the risk of KOA. In the study of Qing yu and colleagues, BMIs were higher in the group with KOA than in the group without KOA, which is aligned with the current research [6]. Some studies consider female gender as one of the risk factors for knee pain and KOA [18–20]. It was seen in the present study that the prevalence of musculoskeletal disorders is higher in women than men. Of course, it is noteworthy that the number of women participating in the study was more than men, which may be due to men's employment and business when the bus was accepting patients and their lack of opportunity to visit the bus.

The study results of the Haq and colleagues also indicated a high prevalence of osteoarthritis among women [13]. The study by Zeng and colleagues in China showed that the prevalence of KOA was higher among women than men [6]. Research results in Australia also indicated that the prevalence of osteoarthritis among women is higher than men [21].

Studies have revealed that some occupational physical activities can increase the risk of osteoarthritis [22–24]. In the present study, knee pain was higher among housewives than other participants in the study. The findings of Dahaghin and colleagues study also indicated that housewives are more prone to KOA than women working outside [25].

Of course, less muscle mass in women than men can also have more impact on their functional limitations along with aging [26] that can lead to a high prevalence of knee pain among housewives, which needs more investigations.

The study conducted by Ricci and colleagues showed that arthritis is prevalent in workers aged 40–65. The findings of our study also showed that after housekeeping, workers had the most experience with knee pain among other studied occupations. However, knee pain experience was for less than half of the workers. Of course, this may be due to the small number of workers participating in the study [24].

## 4. Conclusions

The prevalence of knee pain in the population over the age of 50 who participated in the study was high. Knee pain and stiffness were more prevalent in women than men. The high prevalence of this problem was recognized among housewives and subjects with BMI higher than 30.

## Figures and Tables

**Table 1:** Demographic characteristics of the participants in the study.

Gender		
Female	423	71.8%
Male	166	28.2%

Education		
Uneducated	226	38.4%
High school	211	35.8%
Diploma	105	17.8%
Associate	23	3.9%
Bachelor	21	3.6%
Master	3	0.5%
Total	589	100%

Height		
137–147	25	4.2%
147–157	193	32.8
157–167	230	39.0%
167–177	100	17.0%
177–188	32	5.4%

BMI		
<18.5	9	1.6%
18.5–25	126	21.7%
25–30	242	41.7%
>30	203	35.0%

**Table 2:** Results of Section 1 of the knee questionnaire.

The amount of pain	Ability to bend the knee completely	Ability to extend the knee completely	Feeling of knee lock when moving	Crepitation when moving	Swelling of the knee
Never	1	4	354	347	413
	0.2%	0.7	60.1%	58.9%	70.1%
Rarely	19	18	81	50	55
	3.2%	3.1%	13.8%	8.5%	9.3%
Some of the time	32	35	76	75	49
	5.4%	5.9%	12.9%	12.7%	8.3%
Most of the time	84	83	63	98	52
	14.3%	14.1%	10.8%	16.6%	8.8%
All of the time	453	449	14	19	20
	76.9%	76.2%	2.4%	3.2%	3.4%
Total	589	589	588	589	589
	100%	100%	100%	100%	100%

**Table 3:** The prevalence rate of knee pain when doing various activities.

The amount of pain	Standing upright	Sitting or lying	At night when in bed	Up and down the stairs	Walking on a flat surface	Bending the knee	Knee extension	Rotate on the knee
None	371	383	366	260	375	430	421	48
	63.0%	65.0%	62.1%	44.1%	63.2%	73.0%	71.5%	71.0%
Mild	108	101	78	120	97	83	94	91
	18.3%	17.1%	13.2%	20.4%	16.5%	14.1%	16.0%	15.4%
Moderate	58	62	63	97	75	46	42	40
	9.8%	10.5%	10.7%	16.5%	12.7%	7.8%	7.1%	6.8%
Severe	46	37	76	104	39	26	27	36
	7.8%	6.3%	12.9%	17.7%	6.6%	4.4%	4.6%	6.1%
Extreme	6	6	6	8	5	4	5	4
	1.0%	1.0%	1.0%	1.4%	0.8%	0.7%	0.8%	0.7%

**Table 4:** Determination of prevalence of joint stiffness at different times.

Level of stiffness	How severe is your stiffness first after awakening in the morning?	How severe is your stiffness after sitting, lying, or resting later in the day?
None	393	383
	66.7%	65.0%
Mild	81	96
	13.8%	16.3%
Moderate	61	66
	10.4%	11.2
Severe	49	39
	8.3%	6.6%
Extreme	5	5
	0.8	0.8%

**Table 5:** The prevalence of knee exhaustion side effects based on the gender.

		Never	Rarely	Some of the time	Often	Always	Sum
Female	Swelling on the knee	265	47	46	47	18	423
		62.6%	11.1%	10.9%	11.1%	4.3%	100%
	Crepitation when moving	214	39	61	92	17	423
		50.6%	9.2%	14.4%	21.7%	4.0%	100%
	The problem of knee when moving	222	67	63	59	11	423
		52.5%	15.8%	14.9%	13.9%	2.6%	100%
	Ability to extend the knee completely	3	15	32	68	305	423
		0.7%	3.5%	7.6%	16.1%	72.1%	100%
	Ability to bend the knee completely	1	16	29	69	308	423
		0.2%	3.8%	6.9%	16.3%	72.8%	100%

Gender	Swelling on the knee	148	8	3	5	2	166
		89.2%	4.8%	1.8%	3.0%	1.2%	100%
	Crepitation when moving	133	11	14	6	2	166
		80.1%	6.6%	8.4%	3.6%	1.2%	100%
	The problem of knee when moving	132	14	13	4	3	166
		79.5%	8.4%	7.8%	2.4%	1.8%	100%

Male	Ability to extend the knee completely	1	3	3	15	144	166
		0.6%	1.8%	1.8%	9.0%	86.7%	100%
	Ability to bend the knee completely	0	3	3	15	145	166
		0%	1.8%	1.8%	9.0%	87.3%	100%

**Table 6:** The prevalence of gender-based knee pain in the studied participants.

		No pain	Mild	Moderate	Severe	Extreme	Total
Female	Rotate on the knee	129	81	55	100	100	423
		30.5%	19.1%	13.0%	23.6%	23.6%	100%
	Knee extension	275	78	35	31	31	423
		65.0%	18.4%	8.3%	7.3%	7.3%	100%
	Walking on a flat surface	278	81	37	23	23	423
		65.7%	19.1%	8.7%	5.4%	5.4%	100%
	Going up or down stairs	284	71	42	22	22	423
		67.1%	16.8%	9.9%	5.2%	5.2%	100%
	At night while in bed	239	82	62	35	35	423
		56.5%	19.4%	14.7%	8.3%	8.3%	100%
	Sitting or lying	148	95	83	89	89	423
		35.0%	22.5%	19.6%	21.0%	21.0%	100%
	Standing upright	232	66	54	65	65	423
		54.8%	15.6%	12.8%	15.4%	15.4%	100%

Gender	Rotate on the knee	102	17	24	20	20	166
		61.4%	10.2%	14.5%	12.0%	12.0%	100%
	Knee extension	143	13	5	5	5	166
		86.1%	7.8%	3.0%	3.0%	3.0%	100%
	Walking on a flat surface	143	13	5	4	4	166
		86.1%	7.8%	3.0%	2.4%	2.4%	100%

Male	Going up or down stairs	146	12	4	4	4	166
		88.0%	7.2%	2.4%	2.4%	2.4%	100%
	At night while in bed	133	15	13	4	4	166
		80.1%	9.0%	7.8%	2.4%	2.4%	100%
	Sitting or lying	112	25	14	15	15	166
		67.5%	15.1%	8.4%	9.0%	9.0%	100%
	Standing upright	134	12	9	11	11	166
		80.7%	7.2%	5.4%	6.6%	6.6%	100%

**Table 7:** The prevalence of knee pain associated with the BMI of the studied participants.

Question	Answer																			
	BMI < 18.5					18.5–25					25–30					BMI > 30				
	No pain	Mild	Moderate	Severe	Extreme	No pain	Mild	Moderate	Severe	Extreme	No pain	Mild	Moderate	Severe	Extreme	No pain	Mild	Moderate	Severe	Extreme
Rotate on the knee	7	1	1	0	0	101	11	6	7	1	18	33	19	9	1	126	43	14	19	1
	1.2%	0.2%	0.2%	0%	0%	17.4%	1.9%	1.0%	1.2%	0.2%	31.0%	5.7%	3.3%	1.6%	0.2%	21.7%	7.4%	2.4%	3.3%	0.2%
Knee extension	7	1	1	0	0	101	12	7	5	1	179	35	20	6	2	130	43	14	15	1
	1.2%	0.2%	0.2%	0%	0%	17.4%	2.1%	1.2%	0.9%	0.2%	30.9%	6.0%	3.4%	1.0%	0.3%	22.4%	7.4%	2.4%	2.6%	0.2%
Bending the knee	7	1	1	0	0	101	12	7	5	1	184	28	22	7	1	133	40	16	13	1
	1.2%	0.2%	0.2%	0%	0%	17.4%	2.1%	1.2%	0.9%	0.2%	31.7%	4.8%	3.8%	1.2%	0.2%	22.9%	6.9%	2.8%	2.2%	0.2%
Walking on a flat surface	7	0	2	0	0	85	18	12	10	1	166	33	30	10	2	111	44	29	18	1
	1.2%	0%	0.3%	0%	0%	14.7%	3.1%	2.1%	1.7%	0.2%	28.6%	5.7%	5.2%	1.7%	0.3%	19.1%	7.6%	5.0%	3.1%	0.2%
Up and down the stairs	4	2	2	1	0	68	19	15	22	2	111	42	44	35	2	67	54	35	44	3
	0.7%	0.3%	0.3%	0.2%	0%	11.7%	3.3%	2.6%	3.8%	0.3%	20.5%	7.2%	7.6%	6.0%	0.3%	11.6%	9.3%	6.0%	7.6%	0.5%
At night when in bed	5	3	0	1	0	84	8	14	19	1	167	26	23	25	1	106	39	25	30	3
	0.9%	0.5%	0%	0.2%	0%	14.5%	1.4%	2.4%	3.3%	0.2%	28.8%	4.5%	4.0%	4.3%	0.2%	18.3%	6.7%	4.3%	5.2%	0.5%
Sitting or lying	6	2	1	0	0%	92	15	11	7	1	164	37	29	11	1	116	45	21	18	3
	1.0%	0.3%	0.2%	0%		15.9%	2.6%	1.9%	1.2%	0.2%	28.3%	6.4%	5.0%	1.9%	0.2%	20.0%	7.8%	3.6%	3.1%	0.5%
Standing upright	8	1	0	0	0	90	15	10	10	1	159	43	27	12	1	110	47	20	23	3
	1.4%	0.2%	0%	0%	0%	15.5%	2.6%	1.7%	1.7%	0.2%	27.4%	7.4%	4.7%	2.1%	0.2%	19.0%	8.1%	3.4%	4.0%	0.5%

## Data Availability

The datasets used and/or analyzed during the current study are available from the corresponding author upon request.
